# Termite Resistant Properties of Sisal Fiberboards

**DOI:** 10.3390/insects2040462

**Published:** 2011-10-31

**Authors:** Firda Aulya Syamani, Bambang Subiyanto, Muhamad Yusram Massijaya

**Affiliations:** 1 RDU for Biomaterials, Indonesian Institute of Sciences, Cibinong 16911, Indonesia; 2 Center for Innovation, Indonesian Institute of Sciences, Jakarta 12710, Indonesia;E-Mail: subyanto@cbn.net.id; 3 Department of Forest Products Technology, Faculty of Forestry, Bogor Agricultural University, Bogor 16680, Indonesia; E-Mail: mymassijaya@yahoo.co.id

**Keywords:** sisal fiberboard, termite-resistance, *Coptotermes gestroi*, JWPA Standard-TW-S.1-1992

## Abstract

A study was carried out to test sisal (*Agave sisalana* Perrine) fiberboards properties as building materials against Asian subterranean termite, *Coptotermes gestroi* (Wasmann). Evaluation was in the laboratory according to the JWPA Standard-TW-S.1-1992. To improve mechanical properties of fiberboards made from sisal fibers, the boards were overlaid by rubber veneer, betung bamboo matting or formica. Result showed that the formica-overlaid sisal fiberboards performed better than other overlaid fiberboards against *C. gestroi*.

## 1. Introduction

Sisal fiberboards bonded by urea formaldehyde (UF), melamine urea formaldehyde (MUF) or phenol formaldehyde (PF) adhesives could meet the requirements of mechanical properties designated in JIS A5908-1994, whereas the physical properties of the boards were not satisfactory, especially thickness swelling properties [1]. The necessity to improve this unsuitability as building materials gave us some ideas of which sisal fibers prepared by a ring flaker were bonded with isocyanate adhesive, followed by overlaying by various materials on both the upper and lower faces of the boards [2]. Sisal fibers processed by a ring flaker significantly improved thickness swelling properties of sisal fiberboards, and the overlaying resulted in better mechanical properties.

Following these improvements, determination of board durability against termites was necessary. The tropical climate is suitable for survival of termites and this has become a threat to human dwellings in urban environments due to their ability to attack wooden building materials.

Subterranean termites are among the most widely distributed wood-attacking insects in Indonesia. Not only in Indonesia, *Coptotermes gestroi* Wasmann is the most destructive species in Thailand, attacking not only buildings and other timber constructions, but also any other cellulosic materials and furniture [3]. Genus *Coptotermes* is most destructive to wooden structures because they can damage wood in a short period [4]. Since *Coptotermes* do not always build their nest in contact with soil, but in infested timbers where occasional water supply such as rain water droplets from a leaky roof is available for termites [5], *Coptotermes gestroi* (Wasmann) is considered as a serious pest in Southeast Asia, causing damage to 63%–90% structures and buildings in Malaysia, Thailand and Singapore. Doors, window frames and parquet floors were infested with termites [6].

This study, therefore, reports the resistance of sisal fiberboards against common Asian subterranean termites, *C. gestroi* determined by the standardized laboratory method.

## 2. Materials and Methods

Two types of sisal fiberboards were prepared for the study: one was bare sisal fiberboards, namely, with no overlayings and the other with overlayings.

### 2.1. Bare Sisal Fiberboards

Sisal fibers were formed into boards by bonding with 8%, 10%, and 12% of isocyanate resin based on an oven-dried weight of sisal fibers. Sisal fibers and isocyanate were blended by a drum mixer and hand-formed before hot-pressed at 0.8 N/mm^2^ and 140 °C for 10 minutes. Target density of 1 cm-thick boards was 0.6 g/cm^3^.

### 2.2. Overlaid Sisal Fiberboards

The sisal fiberboards prepared by the above-mentioned manner were overlayed by rubber wood veneer, betung bamboo matting or formica. The amount of isocyanate was 10% based on an oven-dried weight of sisal fibers. Bonding of sisal fibers was done by 75% of the total isocyanate and the remaining was for bonding overlaying materials on both face and back faces. A single-stage board-hot-pressing was conducted at 0.8 N/mm^2^ and 140 °C for 10 minutes.

### 2.3. Termite-Resistance Test

Forced-feeding termite test was conducted with prepared fiberboards according to the Japan Wood Preserving Association (JWPA) standard No. 12-1992 (renamed as JWPA Standard-TW-S.1-1992) [7], provided that three replications were tested using *Coptotermes gestroi*. An individual test specimen of sisal fiberboard (20 × 20 × 10 mm) was placed at the center of the plaster bottom of a cylindrical test container (80 mm in diameter, 60 mm in height) with 150 workers and 15 soldiers of *C. gestroi*. The assembled containers were put on dampened cotton pads to supply water to the specimens and kept at 28 °C and 80% relative humidity. Termite mortality was determined regularly for 3 weeks (21 days). The percent mass loss of each test specimen was calculated from the difference in the oven-dried weights before and after the termite test. Data in percentage were subjected to Mean, Standard error and analysis of variance (ANOVA). Means were separated with Tukey's HSD.

## 3. Results and Discussion

### 3.1. Mass Loss

**Figure 1:**
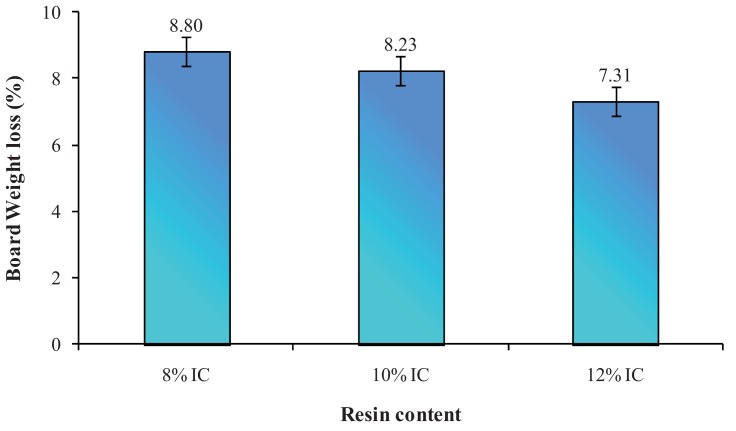
Mean mass loss in percentage of bare sisal fiberboards at three levels of isocyanate contents.

All samples had similar densities, showed no difference at 95% confidence level and the board mass loss was not affected. Sisal fiberboard resin contents affected the mean mass losses of bare sisal fiberboards as shown in Figure 1. Although there was no significant difference in sisal fiberboards mass losses between 10% and 12% isocyanate resin contents, 8% resin content sisal fiberboards showed different mass losses. Mean mass losses were 8.80%, 8.23% and 7.31% at 8%, 10% and 12% resin contents, respectively. The higher resin contents showed less attack by termites, possibly due to the reluctance in feeding activity of termites. One of the advantages of using isocyanate as an adhesive was to improve the durability of wood against deterioration. Visual observation showed that termites first attacked the core part of boards by making entry holes into the fiberboards. This behavior is a manisfestation of termite's characteristics that is cryptobiotic; termites tend to hide themselves and avoid the light [8]. Hardness gradients induced by manufacturing process might account for this phenomenon. Surface-layers were solidified during the hot-compression, impacting higher density than the core portion of the board.

A similar attack pattern was seen for overlaid-boards. It has been commonly known that termites attack the wood to get nutrition, i.e. cellulose. The amount of cellulose in sisal fibers ranges between 67%–78% [9], while betung bamboo contains approximately 52.9% [10]. In addition, the hardness of bamboo surface due to silica content, obstruct termite attack. Therefore, termites attack the core layer first, and then attack overlaying material of betung bamboo matting.

**Figure 2:**
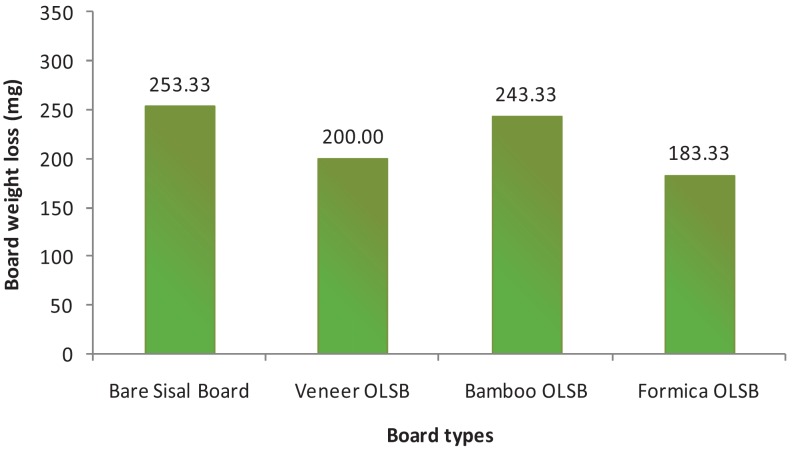
Mean mass loss in mg of bare and overlaid sisal fiberboards. OLSB: overlaid sisal fiberboard.

Among overlaid sisal fiberboards, the mass loss of bamboo-matting overlaid-sisal fiberboard was (243.33 mg in average) higher than rubber-veneer-overlaid sisal fiberboards or formica-overlaid sisal fiberboards (Figure 2). Bamboo is susceptible to microorganisms and insects and whole bamboo in the dry state can be attacked by *Dinoderus* sp. borers and drywood termites [10], due to high content of starch and cellulose. The mass loss of sisal fiberboards overlaid by rubber veneer was high, recording 200 mg on average, as a result of rubber susceptibility to biodegrading agents and low durability as categorized in class V [11].

**Figure 3:**
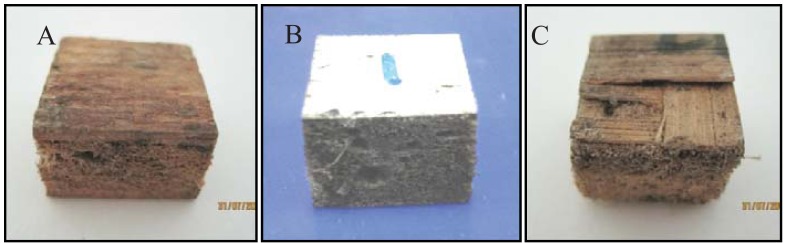
Termite attack on sisal board with rubber wood veneer overlaid (**A**), formica overlaid (**B**) and betung bamboo matting overlaid (**C**) [12].

The type of overlaying materials did not affect sisal fiberboard weight loss and showed no significant difference at 95% confidence level. Visual observation showed that termite attacked the core parts of the board as a manisfestation of cryptobiotic characteristic. The termite attack on the formica-overlaid sisal fiberboards was lower than on rubber veneer or bamboo matting overlaid sisal fiberboards, although the amount of sisal fibers in formica-overlaid sisal fiberboard was higher than in sisal fiberboard with rubber veneer or bamboo matting overlaying. The density of formica (1.17 g/cm^3^) was higher than rubber veneer and betung bamboo matting, 0.57 g/cm^3^ and 0.53 g/cm^3^, respectively. Formica surface was impregnated with melamine, producing a hard surface. Therefore, termite did not attack formica but attacked only the core parts of the board. While in contrast, rubber veneer and bamboo matting are susceptible to termite attack. Sisal fiberboards with rubber veneer and bamboo overlaying weight loss were higher because termites attack the overlaying materials and also sisal fiber at the core part of the boards. Figure 3 shows termite attack on sisal fiberboards with various overlaying.

### 3.2. Termite Mortality

**Figure 4:**
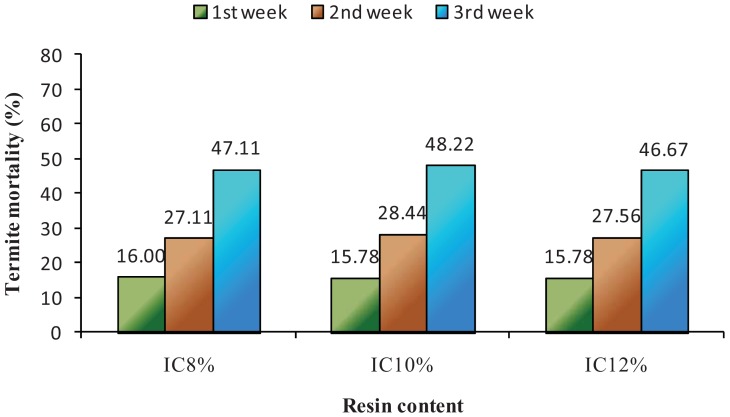
Weekly termite mortality on bare sisal fiberboards at three isocyanate contents.

**Figure 5:**
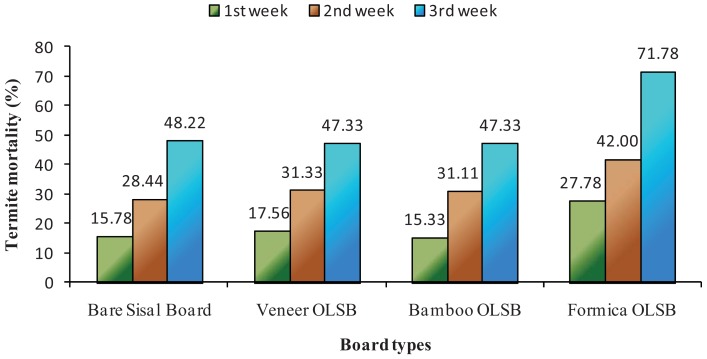
Weekly termite mortality on overlaid sisal fiberboards.

Figures 4 and 5 show the weekly termite mortality on bare and overlaid sisal fiberboards, respectively. Sisal fiberboard resin contents did not significantly affect termite mortality (Figure 4). However, the type of overlaying materials showed significant termite mortality, different at 95% confidence level. Formica-overlaid sisal fiberboards showed the highest mortality and proved significantly different from rubber-veneer-overlaid sisal fiberboards or bamboo matting overlaid sisal fiberboard (Figure 5).

Since formica consisted of several layers of kraft paper impregnated with resin protected with hard surface of melamine after curing by heat, its solidified hardness may have played a part in repelling termites. With limited food source which could not supply termite colonies with sufficient nutrition for survival, termites often feed on their non-productive colony members [13].

## 4. Conclusions

The sisal fiberboards bonded at higher isocyanate contents were less attacked by *Coptotermes gestoi*. Termites attacked the core parts of the board as a manifestation of cryptobiotic characteristics. The type of overlaying materials did not affect sisal fiberboard weight loss due to termite attack, although formica-overlaid sisal fiberboards which has a relatively hard surface, over-performed compared to rubber veneer or bamboo matting overlaid sisal fiberboards. These facts appear to suggest that utilization of hard surface materials and modified sisal fiber would improve sisal fiberboard durability against termite attack.
